# Feasible metabolisms in high pH springs of the Philippines

**DOI:** 10.3389/fmicb.2015.00010

**Published:** 2015-02-10

**Authors:** Dawn Cardace, D'Arcy R. Meyer-Dombard, Kristin M. Woycheese, Carlo A. Arcilla

**Affiliations:** ^1^Microbe Mineral Interactions Laboratory, Department of Geosciences, University of Rhode IslandKingston, RI, USA; ^2^Ecosystem Dynamics in Geochemical Environments Lab, Department of Earth and Environmental Science, University of Illinois at ChicagoChicago, IL, USA; ^3^National Institute of Geological Sciences, College of Science, University of the PhilippinesDiliman, Quezon City, Philippines

**Keywords:** serpentinization, Philippines Islands, high pH springs, ultramafic, geochemical modeling, microbial metabolisms

## Abstract

A field campaign targeting high pH, H_2_-, and CH_4_-emitting serpentinite-associated springs in the Zambales and Palawan Ophiolites of the Philippines was conducted in 2012-2013, and enabled description of several springs sourced in altered pillow basalts, gabbros, and peridotites. We combine field observations of pH, temperature, conductivity, dissolved oxygen, and oxidation-reduction potential with analyses of major ions, dissolved inorganic carbon, dissolved organic carbon, and dissolved gas phases in order to model the activities of selected phases important to microbial metabolism, and to rank feasible metabolic reactions based on energy yield. We document changing geochemical inventories in these springs between sampling years, and examine how the environment supports or prevents the function of certain microbial metabolisms. In all, this geochemistry-based assessment of feasible metabolisms indicates methane cycling, hydrogen oxidation, some iron and sulfur metabolisms, and ammonia oxidation are feasible reactions in this continental site of serpentinization.

## Introduction

Scientific understanding of biosphere-geosphere interactions in Earth's deep subsurface habitats is incomplete. In general, it has been estimated that this deep biosphere could accommodate up to 2 × 10^14^ tons of biomass, which would represent more biomass than on Earth's surface (Gold, 1992; Whitman et al., 1998); more recently, this biomass has been estimated as far less, based on data from diverse subseafloor habitats (Jorgensen, 2012; Kallmeyer et al., 2012). The impact that this reservoir of biomass has on biogeochemical cycling on and in Earth is little known.

Recent characterizations of seabed vents and continental springs hosted in ultramafic (i.e., very Fe- and Mg-rich) rocks provided new insight into a previously little described aspect of the deep biosphere (see site summaries and references in Table 1). The mineralogy of these rocks differentiates them from classic black or white smoker systems in the seabed and similar hydrothermal systems on land. Whereas the chemistry and microbial ecology associated with black and white smoker systems are tied to the reaction of circulating seawater with minerals of the oceanic crust, ultramafic minerals constitute the deeper, mantle-type rock unit, with correspondingly higher proportions of the mineral olivine. The reaction of seawater and/or meteoric water with olivine drives mineral transformations in the ultramafic regions of the seabed and continental subsurface, generating chemical energy that has the potential to sustain hidden microbial ecosystems that extend beneath seabed vents and continental springs.

**Table 1:** **Summary of sites of ongoing research into the geobiology of ultramafic rocks**.

**Setting**	**Geographic Location**	**References**
Submarine seeps/vents	Lost City Hydrothermal Field (LCHF) near the Mid-Atlantic Ridge (MAR), ~40°N	Kelley et al., 2001; c.f., Früh-Green et al., 2003; Schrenk et al., 2004; Kelley et al., 2005; Brazelton et al., 2006, 2011
	Rainbow and Logatchev Hydrothermal Fields	Charlou et al., 1998, 2002; Bach et al., 2004; Schmidt et al., 2007; Konn et al., 2009
	Saldanha Massif	Dias and Barriga, 2006; Dias et al., 2011
	Kairei Vent Field, Central Indian Ridge	Gallant and Von Damm, 2006; Kumagai et al., 2008; Nakamura et al., 2009; Wang et al., 2012
Continental springs	Northern California and Oregon (USA)	Barnes et al., 1967, 1972, 1978
	Coast Range Ophiolite Microbial Observatory (CROMO), Lake County, CA (USA)	Cardace et al., 2013
	Del Puerto Ophiolite seeps, CA (USA)	Blank et al., 2009
	The Cedars ultrabasic springs, Sonoma County, CA (USA)	Morrill et al., 2013; Suzuki et al., 2013, 2014
	Oman	Barnes et al., 1978; Paukert et al., 2012; Boulart et al., 2013; Chavagnac et al., 2013a,b
	New Caledonia	Barnes et al., 1978; Monnin et al., 2014
	Bosnia	Barnes et al., 1978
	Newfoundland (Canada)	Brazelton et al., 2013; Szponar et al., 2013
	Italy	Bruni et al., 2002; Cipolli et al., 2004; Boschetti and Toscani, 2008; Boulart et al., 2013; Chavagnac et al., 2013a,b
	Portugal	Marques et al., 2008; Tiago and Verissimo, 2013
	Costa Rica	Sanchez-Murillo et al., 2014
	Norway	Okland et al., 2012; Daae et al., 2013
	Turkey	Hosgormez et al., 2008; Etiope et al., 2011; Meyer-Dombard et al., 2015

With the addition of water, fayalite (iron-rich olivine, Fe_2_SiO_4_), forsterite (magnesium-rich olivine, Mg_2_SiO_4_), and pyroxene [here taken as (Na,Ca) (Mg,Fe,Al)Si_2_O_6_] in ultramafic rocks can react to form serpentine minerals (e.g., lizardite, chrysotile, and antigorite), hydroxides, and associated phases (Prichard, 1979), as shown in reaction (1).

(1) $$\begin{array}{l} {\text{Fe}}_{2}{\text{SiO}}_{4}+5{\text{Mg}}_{2}{\text{SiO}}_{4}+9{\text{H}}_{2}\text{O}\to 3{\text{Mg}}_{3}{\text{Si}}_{2}{\text{O}}_{5}{\left(\text{OH}\right)}_{4}\\ \;+\text{ Mg}{\left(\text{OH}\right)}_{2}+2\text{Fe}{\left(\text{OH}\right)}_{2}\\ \text{ Fayalite}+\text{forsterite}+\text{water}\to \text{serpentine}+\text{brucite}\\ \;+\text{ iron hydroxide} \end{array}$$

During dissolution of minerals in mafic and ultramafic rocks, protons (H^+^) are consumed, and eventually the dominant hydroxide ions drive up the pH of the aqueous system, producing the very high pH values (>11) observed in actively serpentinizing systems. Concurrently, hydrogen is generated from reaction of the rock assemblage, when Fe^2+^ in Fe(OH)_2_ from (1) is oxidized to magnetite, coupled to the reduction of water, as in reaction (2).

(2) $$3\text{Fe}{\left(\text{OH}\right)}_{2}\to {\text{Fe}}_{3}{\text{O}}_{4}{+\text{2H}}_{2}{\text{O}+\text{H}}_{2}$$

H_2_ is a well described and well utilized fuel for biological metabolism (Jannasch and Mottl, 1985; Orphan and Hoehler, 2011; Petersen et al., 2011). The H_2_ yield from serpentinization is a critical driver of microbial ecosystems in ultramafic-associated environments (Sleep et al., 2004; Cardace and Hoehler, 2009; McCollom and Seewald, 2013; Schrenk et al., 2013), with compelling relevance also to life on the Early Earth (Russell et al., 2010, 2013) and astrobiology (Schulte et al., 2006; Ehlmann et al., 2011; Hellevang et al., 2011; Chassefiere et al., 2013; Lammer et al., 2013; Viviano et al., 2013). The total yield of H_2_ is constrained by parent rock characteristics such as Fe content and partitioning (McCollom and Bach, 2009; Klein et al., 2009). H_2_ can be used in chemotrophic energy production for fixation of CO_2_ into biomass by a diverse range of microorganisms, and is thus has excellent life-supporting potential in environments lacking light or abundant organic matter. Many H_2_-dependent metabolisms deliver large bioenergetic yields (Amend and Shock, 2001; Amend et al., 2011), and several deep biosphere environments on Earth are postulated to have metabolic chains dependent on similar lithogenic H_2_ (Chapelle et al., 2002; Nealson et al., 2005; D'Hondt, 2013).

Methane, formate, and modest amounts of acetate are all known geochemical products of serpentinization (Russell et al., 2010) and are likely important to the deep biosphere of serpentinites. In zones of active serpentinitzation in the deep seabed, for example, CO_2_, supplied by hydrologic connection to the global ocean, is thought to be reduced by H_2_, yielding formate and methanol (Seewald et al., 2006). Reducing the compounds further to produce methane or higher hydrocarbons likely requires metal alloy catalysts (Horita and Berndt, 1999; Proskurowski et al., 2008; Russell et al., 2010; Etiope and Ionescu, 2014). In fact, chemolithoautotrophy in mixing zones between ultramafic rock-hosted hydrothermal systems and seawater has been supported by geochemical modeling; hydrogen oxidation, methanotrophy, sulfate reduction, and methanogenesis are all feasible given this environment (McCollom, 2007).

Additionally, active serpentinization of ultramafic rocks creates distinctive geochemical characteristics in related formation fluids and groundwaters, conventionally sorted by major ion chemistry into Type I and Type II waters (Neal and Stanger, 1984, 1985). Type I waters are produced when meteoric water infiltrates shallow aquifers of ultramafic rock in a so-called open system, that has incoming, ample dissolved CO_2_ derived from the atmosphere; Mg^2+^ from country rock is the major cation, and HCO^−^_3_ is the major anion, and these are also titled Mg^2+^-HCO^−^_3_ waters. Type II waters are produced when Type I waters are isolated from/closed to the atmosphere, continue to react with country rock, and deposit stable Mg-rich secondary minerals such as serpentine, hydroxides, and some carbonates while Ca^2+^ remains dissolved; residual Ca^2+^ from country rock is the major cation, and the loss of protons (they are taken up by country rock) leaves a high activity of OH^−^, and these are termed Ca^2+^-OH^−^ waters. Globally, land-based springs sourced in ultramafic rocks exhibit a range in pH values and ion chemistry, with pH values up to >12 and Ca:Mg ratios up to ~600:1 (c.f., Barnes et al., 1967, 1978).

In this work, we characterize the aqueous geochemistry of springs sourced in mafic-ultramafic units of the Zambales and Palawan Ophiolites (tectonically uplifted oceanic lithosphere, comprising mantle rocks, oceanic crust, and marine sediments) in the Philippines, and integrate these data into a model of feasible, co-occurring metabolic strategies that can be supported, based on the geochemistry of these systems. New microbiological findings related to Philippines high pH springs are coupled to the work described here (Woycheese et al., 2015).

## Geologic setting

The Philippines archipelago straddles a complex set of tectonically dissected microplates sandwiched between the Manila and Philippine Trenches, driven by the meeting of the Philippine and Eurasian tectonic plates. The Zambales Ophiolite in western Luzon features a well preserved ophiolite assemblage exposing mantle peridotite, deep crustal gabbro, ocean crust pillow basalts that solidified on the seafloor, and marine sedimentary formations (Evans and Hawkins, 1989). This ophiolite complex has an area of ~160 km by 40 km, with clearly recognizable units of Eocene Age oceanic crust and mantle-derived rocks, thought to have been emplaced in the Oligocene to Early Miocene, based on stratigraphic studies (Aurelio and Pena, 2010). Cretaceous age rocks of the Palawan Ophiolite crop out in the vicinity of San Isidro Spring (SI1) and Mainit Falls Spring (MF1), comprising a heavily tectonized, 30 km by 300 km swath of uplifted oceanic crust, likely emplaced in the Eocene, with extensive outcrops of seafloor rock types, such as sandstones, cherts, basalts, gabbros, and both olivine- and pyroxene-dominated mantle units (Aurelio and Pena, 2010).

Though gas-rich springs in Palawan are nearly undescribed in the literature (minor mention in Giggenbach and Poreda, 1993), previous work has suggested active serpentinization in Zambales Ophiolite ultramafic rocks in particular. For Zambales, concentrations and isotopic compositions of gases emanating from various related springs and seeps are consistent with a serpentinization origin: gases measured at several seeps at the Los Fuegos Eternos location in the Zambales region by Abrajano et al. (1988) indicate a CH_4_:H_2_ ratio of 55:42 in a dry gas seep and 13:8 in a water saturated sediment–the rest being an unresolved mixture of N_2_ and CO, with the systems extremely low in CO_2_ as expected (Abrajano et al., 1988).

## Materials and methods

### Sampling

**Figure 1:**
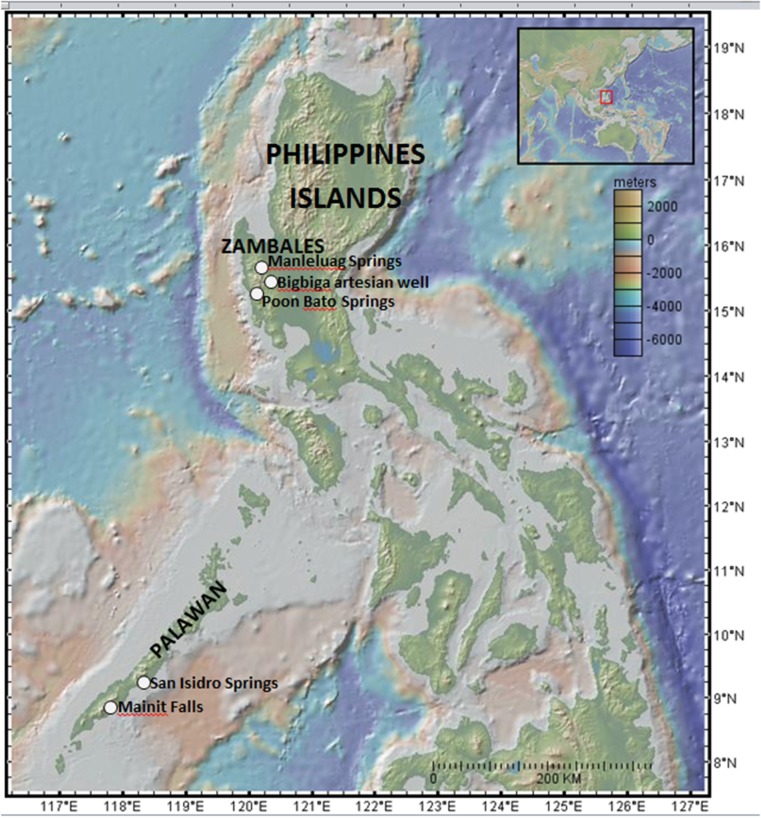
**Elevation map shows the Philippines, including Palawan, with sampling localities indicated**. Inset provides larger scale view of the Western Pacific, with red box showing location of main map. Image generated using GeoMapApp (Ryan et al., 2009): Marine Geoscience Data System (MGDS; www.marine-geo.org).

**Figure 2:**
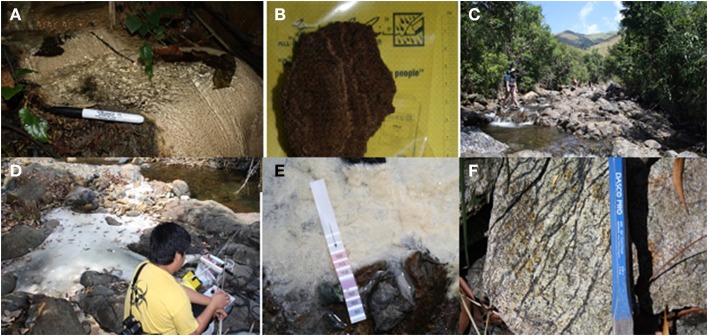
**(A)** Active flowstone deposit near Manleluag Spring; terraces of travertine continued into/under the lushly vegetated adjacent landscape. **(B)** Within Manleluag Spring area, soft new travertine deposit. **(C)** Along the Poon Bato River. **(D)** Deeply sourced spring along Poon Bato River(Site PB2), very basic, very reducing, and depositing travertine. **(E)** pH strip showing PB2 edge travertine surface with pH ~11. **(F)** Serpentinite river cobble on the approach to Poon Bato River area springs.

Serpentinite-associated waters were collected for geochemical analysis from 7 sites in the Zambales Ophiolite, ranging from artesian wells to travertine-depositing springs, and 2 springs in the Palawan Ophiolite. Sampling sites are identified in Figure 1; additional detail is provided in Figure 2. A YSI556 Multiparameter System was used to measure simultaneously in the field dissolved oxygen, pH, conductivity, temperature, and oxidation-reduction potential. A portable field spectrophotometer (HACH DR 2800) with commercially available reagent ampules enabled quantification of redox-sensitive chemical species (e.g., nitrate, nitrite, sulfide, ammonia). Water samples for laboratory analysis were collected with triple-flushed syringes (60-ml volumes with luer-lok tips; BD Medical No. 309653), and filtered through syringe filters or through 0.22 μm pore size Millipore Sterivex PVDF filters, assisted by peristaltic pump. Filtered solutions dedicated for ion chromatography analyses were stored cold in clean, triple-rinsed Nalgene bottles, while filtered waters dedicated for elemental analysis were stored in clean, acid-washed sampling bottles (caps were parafilmed in the field to minimize evaporation), and acidified with nitric acid for a final concentration of ~2% nitric acid.

### Aqueous geochemistry

For the 2012 field season, ions were characterized at Arizona State University (ASU). Samples for analysis of cations and anions were filtered into 60 ml Nalgene bottles prepared by soaking overnight in an acid bath. Bottles were kept frozen until analysis. Samples for major ions were filtered in the field through a series of 0.8 and 0.2 μm pore size filters (such as Supor filters made of hydrophilic polyether-sulfone, Pall Scientific). As quickly as possible after sampling, major cation (Na^+^, K^+^, Ca^+2^, Mg^+2^), and anion (SO^−2^_4_, Cl^−^, Br^−^, F^−^) concentrations were measured in the laboratory using ion chromatography (anions: Dionex IonPac AS11 analytical and IonPac AG11 guard columns; cations: Dionex IonPac CS12A analytical and IonPac SG11 guard columns; conductivity detection). Analytical uncertainties are on the order of 5%.

For the 2013 field season, concentrations of some major anions (chloride, bromide, nitrite, nitrate, phosphate, sulfate) were measured with a Dionex DX-120 ion chromatograph (University of Rhode Island, Kingston RI) outfitted with an IonPac AS22 Analytical Column (4 × 250 mm) and IonPac AG22 Guard Column (4 × 50 mm). A 5 point calibration was based on analysis of gravimetrically prepared calibration standards; calibration proved linear with *r*^2^ ≥ 0.999 for all anions tested. Accuracy of data for Cl^−^ is ±3%, for NO^−^_2_ is ±5%, Br^−^ is ±20%, NO^−^_3_ is ±1%, PO^3−^_4_ is ±1%, SO^2−^_4_ is ±10%, based on multiple analyses of the Dionex Seven Anion Standard (PN 056933; Lot Number 20-25VY).

Elemental concentration data were collected with a Thermo Scientific iCAP Q Inductively Coupled Plasma Mass Spectrometer (housed in the Rhode Island IDeA Network for Excellence in Biomedical Research Lab). Calibration was made using the Ultra Scientific ICP-MS calibration standard #2 (Cat.#: IMS102, Ultra Scientific, North Kingstown, RI), presented to the instrument at concentrations of 100, 500, and 1000 ppb in 2% nitric acid. Linear calibration across this concentration range was observed with *r*^2^ ≥ 0.999 for all analytes except Fe(57), for which *r*^2^ ≥ 0.9875. An external reference solution (ICP-SS-50, High Purity Standards, Charleston, SC) was run as an unknown to assess accuracy, which differed by analyte. Uncertainty was observed as ±10% for Na(23), Mg(24), Al(27), K(39), V(51), Cr(52), Mn(55), Fe(57), Co(59), Ni(60), Cu(63), As(75), Se(82), Sr(88); Ca(44) data carry a larger uncertainty of ± 30%. Precision was typically <1%, as shown by very low relative standard deviations for triplicate analyses.

### Stable isotopes of water

Two milli liter volumes of filtered water were stored in vials with PTFE/silicone screw tops (Fisherbrand cat. no. 03-391-15) and analyzed with a Picarro Water Isotope Analyzer L1102i (Brown University Environmental Chemistry Facilities, Providence, RI). In practice, 2 microliters of liquid water were injected into the vaporizer module, which prepared and sent the sample to the laser cavity for measurement. Each sample was measured in triplicate and corrected for memory and drift (using the protocols of Vaughan and Claymore, INSTARR, Univ. of Colorado). All samples were measured with respect to internal lab standards, which were calibrated directly to VSMOW, SLAP and GISP2. Data are reported here with respect to VSMOW; overall precision and accuracy is better than 0.1‰ and 1.0‰ for δ^18^O and δD, respectively.

### Dissolved inorganic carbon (DIC) and dissolved organic carbon (DOC)

Sampling vials were amber I-CHEM vials. DIC septa were butyl/PTFE; DOC septa were silicone/PTFE. Prior to sampling, DIC bottles and septa were acid-washed (overnight soak in 10% HCl by volume solution, rinsed with high purity water of ~18 MΩ resistivity), then dried and assembled. DOC bottles were combusted at 500°C overnight, septa were rinsed, then bottles were spiked with 100 ul of ASC grade 85% phosphoric acid. In the field, all fluid was filtered. Collection bottle [not sampling bottle] was flushed with sample 3x, as was tubing and syringe. DOC bottles were filled using a filter that had been conditioned with several liters of sample. Both DIC and DOC bottles were filled to the top with no air bubbles and kept at ~20°C until analysis. DOC and DIC concentrations were measured with an OI Analytical Model 1010 Wet Oxidation Total Organic Carbon (TOC) Analyzer at ASU, as in Meyer-Dombard et al. (2015). Fluids were reacted with phosphoric acid (DIC) or sodium persulfate (DOC), and CO_2_ was analyzed by continuous flow into a Thermo Delta^Plus^ Advantage mass spectrometer. Three glycine working standards characterized with USGS40 and USGS41 isotopic reference materials were used that encompass expected isotopic variations (low: δ^13^C = −39.64‰, δ^15^N = 1.35‰; mid: δ^13^C = −8.36‰, δ^15^N = 27.9‰; and high: δ^13^C = 15.67‰, δ^15^N = 51.8‰).

### Gas chemistry

10 mL serum vials and blue butyl stoppers were acid-washed (overnight soak in 10% by volume HCl and rinsed in ~18 MΩ Milli-Q distilled water). Vials and stoppers were allowed to air-dry, assembled, and crimped shut prior to flushing (Wheaton, Millville, NJ, USA). Gas sampling vials were flushed with Ar gas at a rate of 8–10 psi pressure: briefly, a 23 gauge needle was inserted into the sealed vial, connected to a Luer-Lock-equipped tubing (BioRad Laboratories, Inc., Hercules, CA, USA) to the Ar tank. To ensure vials were not over-pressured, a venting needle attached to an open-ended 60 mL syringe filled with 10–15 mL of distilled water suspended above the gassing station to allow excess gas to vent without allowing atmospheric gas back into the vial. Flushed sampling vials were transported to the field, and filled with 5 mL of sample water using the following protocol: samples were collected with a syringe, and air bubbles were forced out of sample volume by depressing the syringe stopper. The sampling syringe was flushed 3X, and samples were collected slowly to avoid the draw of vacuum. Samples were poisoned in the field with an 0.5 mL injection of Hg(NO_3_)_2_ (1.71 g/50 mL) and stored upside-down until analysis. Upon return to the USA, samples were immediately analyzed on a Shimadzu GC-2014 gas chromatograph/mass-spectrometer (GC-MS) equipped with flame ionization and thermal conductivity detectors (FID and TCD, respectively), a Carboxen® 1000 60/80 molecular sieve (Sigma-Aldrich Co., LLC, St. Louis, MO, USA), with a 1/8″ × 15′ column (Shimadzu Corporation, Kyoto, Japan). C1 gases of interest were first methanized (converted to CH_4_), combusted, and detected with the FID. H_2_ and Ar were detected on the TCD. Depending on sample concentration, 200–400 μl of the gas in the sampling vial headspace were injected into the GC-MS column. Calibration curves were constructed using H_2_, CO, CH_4_, and CO_2_ standard gases (Sigma-Aldrich Co., LLC, St. Louis, MO, USA), both during and 6 months after analysis; the calibration curves for all gases analyzed suggested that the GC-MS was stable and results were highly reproducible.

### Geochemical modeling

Gibbs energy (ΔG_r_) calculations were used to determine thermodynamically favorable reactions that can serve as microbial metabolisms supported by the environment under study. Gibbs energy calculations can be used to identify points in temperature-pressure-composition space where metabolisms are most favorable.

Values of ΔG_r_ at the temperature, pressure, and chemical composition at the sampled sites were computed with relation (3):

(3) $$\Delta G_{r}=\Delta G_{r}^{{}^{\circ}}+RTlnQ_{r},$$

where Δ*G*°_*r*_ denotes the standard state Gibbs energy values at the temperature and pressure of interest (Δ*G*°*_r_* for chemical species pertinent to this work are readily available in Amend and Shock, 2001), *R* represents the gas constant, *T* represents temperature in Kelvin, and *Q*_r_ is the activity product.

*Q*_r_, the activity product, can be computed from environmental data as shown in relation (4):

(4) $$Q_{r}=\Pi a_{i}^{\upsilon i,r}$$

where *a_i_* represents the activity of the *i*th species, and Υ*_i_*, *_r_* represents the stoichiometric reaction coefficient. Values of *a_i_* are generated from concentration data (Table 1) and are modified by normalization with reasonable activity coefficients, using the geochemical speciation code EQ3 (Wolery, 1992). In this code, activity coefficients are calculated using a variant (B-dot equation) of the Extended Debye-Hückel activity coefficient formalism (Helgeson, 1969), with reference to the SUPCRT92 (Johnson et al., 1992) thermodynamic database. Total dissolved solids (TDS) data, which must be known to quantify the ionic strength of the solution, were estimated from field conductivity data and included in EQ3 input files (see Supplementary Material). Note that EQ3 provides the equilibrium speciation of co-occurring geochemical components based on the data inputs. If data inputs lack a particular chemical species (e.g., acetate), EQ3 provides a rigorous estimate of its activity in the modeled environment, depending on thermodynamic constraints.

It is worth noting that Δ*G*°*_r_* can be calculated at the appropriate temperature and pressure for the aqueous species and minerals using established equations of state (Helgeson et al., 1978, 1981; Shock and Helgeson, 1988, 1990; Tanger and Helgeson, 1988; Shock et al., 1989, 1992), or computed from data for Δ*G*°*_r_* and Δ*G*°*_i_* at temperatures up to 200°C at P_sat_, available in Amend and Shock (2001). Conventionally, Δ*G*°*_r_* can also be calculated from Gibbs energy of formation values, using relation (5):

(5) $$\Delta G_{r}^{{}^{\circ}}=\Sigma \upsilon_{i,r}\Delta G_{i}^{{}^{\circ}}$$

In which Δ*G*°*_r_* is the standard state Gibbs energy of reaction *r*, Υ*_i,r_* is the stoichiometric reaction coefficient of the *i*th species in reaction *r*, which is negative for reactants and positive for products, and Δ*G*°*_i_* is the standard Gibbs free energy of formation of the *i*th species at the temperature and pressure of interest in reaction *r*.

## Results

### Aqueous geochemistry

#### Zambales ophiolite: Manleluag Springs, Bigbiga Artesian Well, and Poon Bato springs

In Zambales, the Manleluag Springs primary locality (ML1) is in a protected park area, with a bubbling spring housed in a sheltered cistern that feeds a set of community-accessible swimming pools. In adjacent undeveloped land, related spring water is emitted from a natural slope (ML2) and flows downhill through increasingly dense vegetation along a travertine-lined creek bed (ML3, encompasses sites CC1 and CC2 in Woycheese et al., 2015). Waters are of moderate to elevated pH (10–11), temperatures near 34°C, and ORP values of −703 to −245 mV (corresponding to Eh ranging from −503 to −45 mV, since there is a +200 correction required to transform observed ORP values to Eh when using the YSI556 ORP electrode kit) (Table 2). Sampling of country rock in the vicinity indicates gabbroic host rock to an unknown depth.

Table 2**Aqueous geochemistry for ophiolite-associated springs in the Philippines**.

**Table:** 

**Expedition**	**Site**	**T (°C)**	**pH**	**cond (mS/cm)**.	**DO (%)**	**ORP**	**NO^−^_3_ (mg/L)**	**NH^+^_4_ (mg/L)**	**Fe^+^2 (mg/L)**	**Fe total (mg/L)**	**S^−2^ (mg/L)**	**SiO_2_ (μg/L)**	**DIC (mg/L)**	**DIC ppm C**	**DIC δ13C (VPDB) (‰)**	**DOC σ (‰)**	**DOC δ 13C (VPDB) (‰)**	**DOC σ (‰)**
*Zambales, 2012*	Manleluag 1, ML1	**34.4**	**10.9**	**0.315**	**1.5**	**−703**	**4.1**	**bdl**	**bdl**	**bdl**	**1290**	**30.5**	<2	−12.8	0.7	0.8	−26.8	0.5
*Zambales, 2012*	Manleluag 2, ML2	**34.3**	**10.8**	**0.337**	**26.6**	**−355**	**4.3**	**0**	**0**	**bdl**	**1236.7**	**30.3**	<2	−16.5	0.7	0.4	−26.5	–
*Zambales, 2012*	Manleluag 3, ML3	**33.8**	**10.8**	**0.307**	**62.5**	**−245**	**5**	**0.9**	**0**	**bdl**	–	–	<2	−18.7	0.7	0.5	−26.8	–
*Zambales, 2012*	Bigbiga well, BB1	**29.7**	**9.3**	**0.349**	**0.8**	**−146**	**1.7**	–	**bdl**	**bdl**	**1**	**830**	38.6	−13.8	0.3	0.3	−26.3	–
*Zambales, 2012*	Poon Bato 1, PB1	**31.5**	**11.3**	**0.505**	**60**	**−136**	**1.3**	**0**	**0.3**	**bdl**	**1**	**298.3**	<2	−25.4	0.7	0.3	−22.9	–
*Zambales, 2012*	Poon Bato 2, PB2 [star pool]	**27.2**	**9.2**	**0.229**	**32.4**	**−86**	**0.9**	**0**	**bdl**	**bdl**	**1**	**403.3**	6	−17.5	0.7	1.1	−24.4	0.4
*Zambales, 2012*	Poon Bato 2, PB2[square pool]	**26.8**	**10.4**	**0.201**	**38.7**	**−164**	–	–	–	**bdl**	–	–						
*Zambales, 2012*	Poon Bato 2, PB2 [red waterfall]	**27.1**	**9.6**	**0.222**	**32.5**	**−174**	–	–	–	**bdl**	–	–						
*Zambales, 2012*	Poon Bato 3, PB3	**28.6**	**11.3**	**0.606**	**7.9**	**−373**	–	–	–	**bdl**	–	–	–	–	–	0.2	−21.4	–
*Zambales, 2012*	Poon Bato Riv, PBR	**27.9**	**8.6**	**0.179**	**97.7**	**15**	–	–	–	**bdl**	–	–	18.5	−12.1	0.5	0.3	−27.8	1
*Palawan, 2012*	San Isidro Spr, SI1.	**47.7**	**10.5**	**0.516**	**12**	**−265**	**7.5**	**0.1**	**0**	**bdl**	**3796.7**	**120**	3.9	−19.1	0.3	0.2	−26.3	–
*Palawan, 2012*	Mainit Falls, MF1	**40.6**	**9.7**	**0.784**	**10.5**	**−287**	**7.5**	**0.9–1.8**	**bdl**	**bdl**	**1550**	**149.5**	28.1	−15.3	0.9	0.3	−26.4	1
*Zambales, 2013*	Manleluag 1, ML1	**34.4**	**10.9**	**0.387**	**0.2**	**−460**	**4**	**0.1**	**0**	**bdl**	**1323.3**	**30.8**	<2	−15.4	0.5	0.3	−29.6	0.4
*Zambales, 2013*	Manleluag 2, ML2	**34.4**	**10.8**	**0.388**	**0.9**	**−425**	**1.6**	**0.9**	**0**	**bdl**	**1780**	**34.7**	<2	−10.8	2	0.1	−25.5	1
*Zambales, 2013*	Manleluag 3, ML3	**32.6**	**10.3**	**0.27**	**4.3**	**−226**		–	–	**bdl**	**213.3**		4.4	−21.1	0.5	0.7	−29.1	0.4
*Zambales, 2013*	Bigbiga well, BB1	**28**	**7**	**0.428**	**0.6**	**−174**	**1.1**	**bdl**	**0**	**bdl**	**45**	**5.9**	35.6	−10.8	0.5	10.6	−30.2	0.4
*Zambales, 2013*	Poon Bato 1, PB1	**29.7**	**9.6**	**0.232**	**5.5**	**−125**	**1.4**	**bdl**	**0.6**	**bdl**	**21.2**	**bdl**	3	−12.8	2	0.3	−26.8	1
*Zambales, 2013*	Poon Bato 2, PB2	**29.7**	**8.7**	**0.189**	**8.7**	**−97**	**1.5**	**bdl**	**0**	**bdl**	**10.3**	**30.5**	22.5	−13.8	0.5	1.2	−25.8	0.4
*Zambales, 2013*	Poon Bato Riv, PBR	**26.4**	**8.3**	**0.17**	**7.4**								21.3	−8.1	0.5	0.6	−23.6	0.4

**Table:** 

**Expedition**	**Site**	**F^−^ ppm**	**Cl^−^ ppm**	**Br^−^ ppm**	**SO^−2^_4_ ppm**	**PO^−3^_4_ ppm**	**NO^−^_2_ ppm**	**NO^−^_3_ ppm**	**Li^+^ ppm**	**Na^+^ ppm**	**NH^+^_4_ ppm**	**K^+^ ppm**	**Mg^+^2 ppm**	**Ca^+^2 ppm ppb**	**Al ppb**	**V ppb**	**Cr ppb**	**Mn ppb**	**Fe ppb**	**Co ppb**	**Ni ppb**	**As ppb**	**Se ppb**	**Sr ppb**
*Zambales, 2012*	Manleluag 1, ML1	bdl	18	bdl	0.7	bdl	bdl	bdl	bdl	23	bdl	0.2	0	3.1	–	–	–	–	–	–	–	–	–	–
*Zambales, 2012*	Manleluag 2, ML2	bdl	18.7	bdl	0.7	bdl	bdl	bdl	bdl	24.4	bdl	0.2	0	3.9	–	–	–	–	–	–	–	–	–	–
*Zambales, 2012*	Manleluag 3, ML3	bdl	17	bdl	0.8	bdl	bdl	bdl	bdl	22.6	bdl	0.2	0.1	3.3	–	–	–	–	–	–	–	–	–	–
*Zambales, 2012*	Bigbiga well, BB1	0.7	4.4	bdl	47.3	bdl	bdl	bdl	bdl	100.5	bdl	0.5	0	1.5	–	–	–	–	–	–	–	–	–	–
*Zambales, 2012*	Poon Bato 1, PB1	bdl	24	bdl	0.1	bdl	bdl	bdl	bdl	23.9	bdl	1.3	0	52.8	–	–	–	–	–	–	–	–	–	–
*Zambales, 2012*	Poon Bato 2, PB2 [star pool]	0.1	11.3	bdl	0.1	bdl	bdl	bdl	bdl	10.3	bdl	0.6	7.6	8	–	–	–	–	–	–	–	–	–	–
*Zambales, 2012*	Poon Bato 2, PB2[square pool]	–	–	–	–	–	–	–	–	–	–	–	–	–	–	–	–	–	–	–	–	–	–	–
*Zambales, 2012*	Poon Bato 2, PB2 [red waterfall]	–	–	–	–	–	–	–	–	–	–	–	–	–	–	–	–	–	–	–	–	–	–	–
*Zambales, 2012*	Poon Bato 3, PB3	bdl	17.9	bdl	0	bdl	bdl	bdl	bdl	15.6	bdl	0.8	0.2	50.2	–	–	–	–	–	–	–	–	–	–
*Zambales, 2012*	Poon Bato Riv, PBR	–	–	–	–	–	–	–	–	–	–	–	–	–	–	–	–	–	–	–	–	–	–	–
*Palawan, 2012*	San Isidro Spr, SI1.	bdl	57.4	0.2	3.9	bdl	bdl	bdl	bdl	91.6	bdl	0.7	0	3.6	–	–	–	–	–	–	–	–	–	–
*Palawan, 2012*	Mainit Falls, MF1	2.8	228.7	0.7	9.7	bdl	bdl	bdl	0.07	269.7	1.9	4.5	0	2.5	–	–	–	–	–	–	–	–	–	–
*Zambales, 2013*	Manleluag 1, ML1	–	17	bdl	<5	bdl	bdl	bdl	–	*18.5*	–	*0.3*	*0.2*	*8.4*	*270.8*	*bdl*	*bdl*	*bdl*	*38.9*	*bdl*	*bdl*	*3.3*	*0.8*	*20.8*
*Zambales, 2013*	Manleluag 2, ML2	–	16.9	bdl	18.5	bdl	bdl	bdl	–	*18.7*	–	*0.3*	*bdl*	*6*	*254.1*	*bdl*	*bdl*	*bdl*	*26.9*	*bdl*	*bdl*	*2.3*	*0.5*	*19.9*
*Zambales, 2013*	Manleluag 3, ML3	–	9.8	bdl	19.3	bdl	bdl	bdl	–	*19.8*	–	*0.4*	*1.7*	*2.2*	*56.1*	*3.1*	*bdl*	*bdl*	*31.6*	*bdl*	*bdl*	*3.3*	*1.3*	*27.4*
*Zambales, 2013*	Bigbiga well, BB1	–	<5	bdl	46.6	bdl	bdl	bdl	–	*119.9*	–	*0.7*	*0*	*1.1*	*bdl*	*bdl*	*bdl*	*bdl*	*bdl*	*bdl*	*bdl*	*1.3*	*bdl*	*bdl*
*Zambales, 2013*	Poon Bato 1, PB1	–	12.4	bdl	9.6	bdl	bdl	bdl	–	*19.2*	–	*0.5*	*0.1*	*37.2*	*41.6*	*bdl*	*bdl*	*bdl*	*172.2*	*bdl*	*bdl*	*bdl*	*0.6*	*66*
*Zambales, 2013*	Poon Bato 2, PB2	–	10.9	bdl	89.1	bdl	bdl	bdl	–	*14.8*	–	*0.2*	*20.7*	*12.9*	*bdl*	*bdl*	*1.7*	*bdl*	*bdl*	*bdl*	*17.5*	*3*	*bdl*	*12*
*Zambales, 2013*	Poon Bato Riv, PBR	–	7.3	bdl	<5	bdl	bdl	bdl	–	*1.4*	–	*0.1*	*23.3*	*2*	*bdl*	*bdl*	*8.6*	*bdl*	*bdl*	*bdl*	*4.8*	*bdl*	*bdl*	*3.2*

*Environmental parameters temperature, pH, conductivity, ORP, and DO (bolded) were measured during sampling by YSI 556 multiprobe meter while field spectrophotometry tests were completed (bolded). Dissolved inorganic carbon (DIC) and dissolved organic carbon (DOC) data pertain to samples collected at the same location as for other analyses. Stable isotope data for ^13^C are presented in per mil (‰) and, as by convention, relative to the Pee Dee Belemnite carbon isotope standard (vPDB); σ connotes standard deviation. Ion chromatography (IC) data are plain font (ppm). Inductively coupled plasma mass spectrometry (ICPMS) data are italicized (ppb). Note that fluoride data are grayed out; relative standard deviations (RSD) were up to 24%, thus data are informational only*.

^*^Ion chromatography (IC) data were collected in two different labs; 2012 data were collected at ASU (Shock Lab), and 2013 data were collected at URI (Cardace Lab and shared facilities).

The Bigbiga Artesian Well (BB1) is a ~30.5-m-deep artesian well that empties into a cistern. When opened, the cistern fills rapidly, providing perennial water access for the local community. Water is of pH 9.3 and has a temperature of 30°C, with an ORP value of −146 mV (corresponding to Eh ~ +54 mV) (Table 2). Well cores, property of co-author Arcilla, indicate that the base of the borehole is in altered pillow basalts overlain by bentonite, a fine-grained, clay-rich sedimentary formation; this site samples waters interacting with rocks equivalent to the top of the ocean crust.

Multiple springs are also located alongside the Poon Bato River, where associated travertine deposits are short-lived, destroyed/repositioned after small magnitude earthquakes and/or seasonally intense rainfalls. PB1 and PB2 are close to each other (<50 m distant) while PB3 is several km upstream. PB1 exhibited well-developed travertine in both sampling years. PB2 showed some loss in travertine extent between 2012 and 2013. PB3 was well developed in 2012, emanating at low flow rates about 5 m away from the Poon Bato River bank, causing deposition of travertine along a winding ~20-meter-long path, ending at the river; this site was wiped out completely in 2013, with much loss of carbonate mineralization due to chemically aggressive rain waters in the intervening seasonal monsoons, which have been intense in recent years. Waters are of very high pH (>11 at PB1 and PB3), with temperatures ranging from 27 to 32°C, and ORP values of −373 to −86 mV (corresponding to Eh ranging from ~−173 to +114 mV) (Table 2). Sampling of country rock indicates that serpentinite (altered mantle rock) is the host rock.

#### Palawan Ophiolite: San Isidro Spring and Mainit Falls Spring

Site SI1 is influenced by hydrothermally altered waters, bringing the surface water temperature to 47.7°C, with 3 times more Cl^−^ and almost 10 times more Na^+^ than in seawater. Water is of elevated pH (10.5), with ORP observed at -265 mV (corresponding to Eh of ~ −65 mV). Similarly, Site MF1 has numerous small sources of hydrothermally altered waters emptying into a main sampling pool with a temperature of 40.6°C; waters exhibit an order of magnitude more Cl^−^ and Na^+^ than seawater (Table 2). Water is of elevated pH (9.7), with ORP observed at −287 mV (corresponding to Eh of ~ −87 mV) (Table 2).

**Figure 3:**
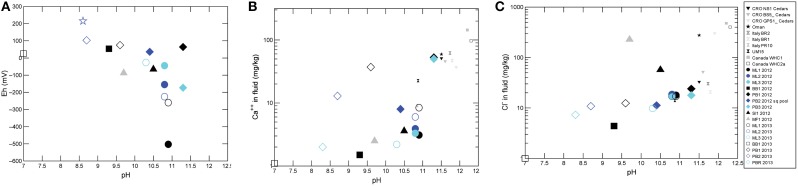
**Aqueous geochemistry data indicate active serpentinization impacting spring waters. (A)** Eh as a function of pH. **(B)** Ca^++^ as a function of pH. **(C)** Cl^−^ as a function of pH for all sites sampled in this study, with other known continental sites of serpentinization for context.

**Figure 4:**
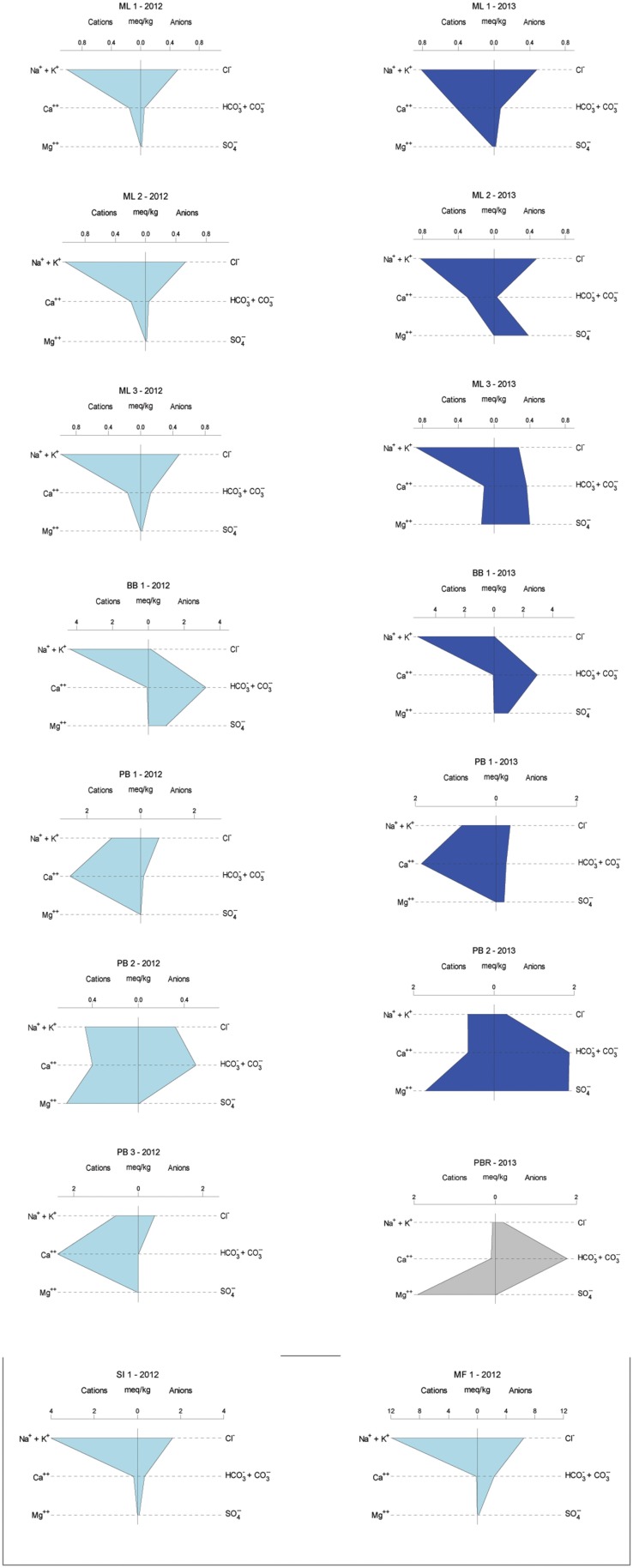
**Geochemical variability across years in the sample set, given as Stiff diagrams, for which similar shapes connote similar major ion patterns**. 2012 samples are in light blue, 2013 samples are in dark blue, and sample sites correspond from left to right except for single visit locations. SI1 and MF1 sites are in Palawan and were visited once. PB3 is the most upstream in the PB series of springs and was completely destroyed by vigorous rains between sampling years. Poon Bato River data are provided for context, in gray.

**Figure 5:**
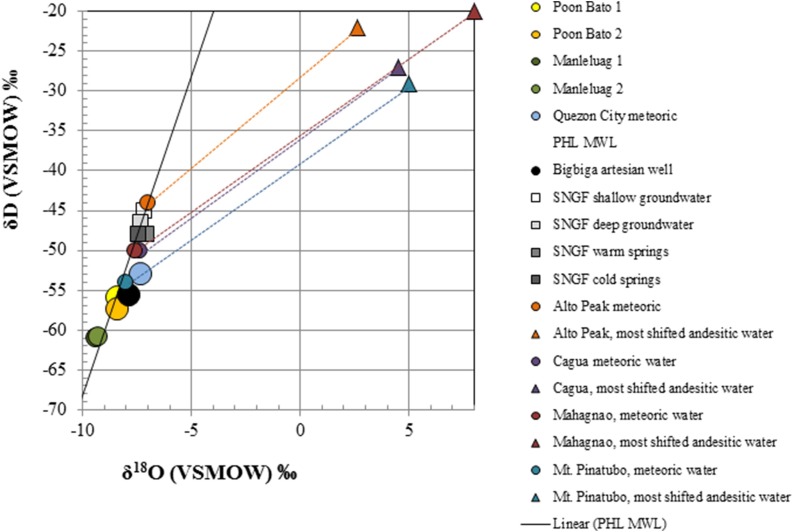
**Stable isotope data for spring waters presented with the current meteoric water line for the Philippines and a suite of meteoric and andesitic waters from the region; the latter are color-coded to show that the source water (circles) are driven to the upper right via interaction with andesitic volcanism (along dashed lines) to the cluster of magmatically associated waters (triangles)**. Note that all of the spring data for this study cluster near/on the MWL for the region, without a significant magmatic water component.

Overall, sampled springs exhibit strong (ML and PB sites) to weak (BB) serpentinization inputs and some hydrothermal inputs, creating a diversity of subsurface aqueous environments. Eh tracks inversely with pH, and Ca^2+^ and Cl^−^ both increase generally with pH (Figure 3). More evocative are the Stiff diagrams provided as a collection of panels in Figure 4, in which the major ion chemistry is charted to provide a geometric field that allows rapid visual identification of similar and different water types. From Figure 4, in terms of dissolved constituents, ML sites, PB sites, and BB1, are 3 distinct water types, and SI1 and MF1 constitute a fourth group. Despite these differences in dissolved constituents, stable isotope data for sampled waters shows that all new data fall near/on the local meteoric water line (LMWL) (see Figure 5, LMWL from Gerardo-Abaya, 2005). The LMWL is shown as a black, positive sloping line, tracking the variation in stable isotopes of oxygen and hydrogen in regional precipitation, as δD is known to have a linear relationship with δ^18^O. When data for spring waters are far from the LMWL, they have experienced isotopic fractionation, perhaps through evaporation or interactions with magmatic processes (note how far from the LMWL are the triangle symbols, Figure 5). When spring waters fall on the LMWL, as shown here in Figure 5, they are taken to be genetically related to regional precipitation. These data are not available for SI1 and MF1, thus their source is unconstrained by this test.

#### Dissolved inorganic and organic carbon (DIC and DOC)

Table 2 presents dissolved inorganic carbon (DIC) data for these springs. ML and PB springs cluster together with SI1 at very low total DIC, with δ^13^C between −10 and −26‰; though not unusual for groundwater values, together these data convey the variability in the DIC of those waters, apparently depleted to some degree with respect to regional Poon Bato River (PBR) samples, for which DIC was observed at ~20 ppm DIC, with δ^13^C between −8 and −12‰. One PB spring data point falls near these surface river waters, suggesting mixing of a deeper, more isotopically light reservoir with surface, river-type waters. BB1 data fall near 40 ppm DIC and between −10 and −15‰ δ^13^C, and may represent waters strongly influenced by continuing metabasalt alteration. MF1, in between identified clusters, may be a mixture of more isotopically depleted water with BB1-type, metabasalt-hosted waters.

Dissolved organic carbon (DOC) data cluster together at <2 ppm DOC and between −22 and −30‰ δ^13^C, indicating a self-consistent DOC inventory that may transcend other site characteristics. The only exception is for BB1 in 2013, for which total DOC is elevated to near 10 ppm; this is likely due to some sample contamination with biofilms near the well outlet. In all cases, DOC is isotopically lighter than DIC, supporting either microbial carbon cycling *in situ* or contributions from another, isotopically very light, source of DOC such as infiltration from surface sources.

### Gas chemistry

**Figure 6:**
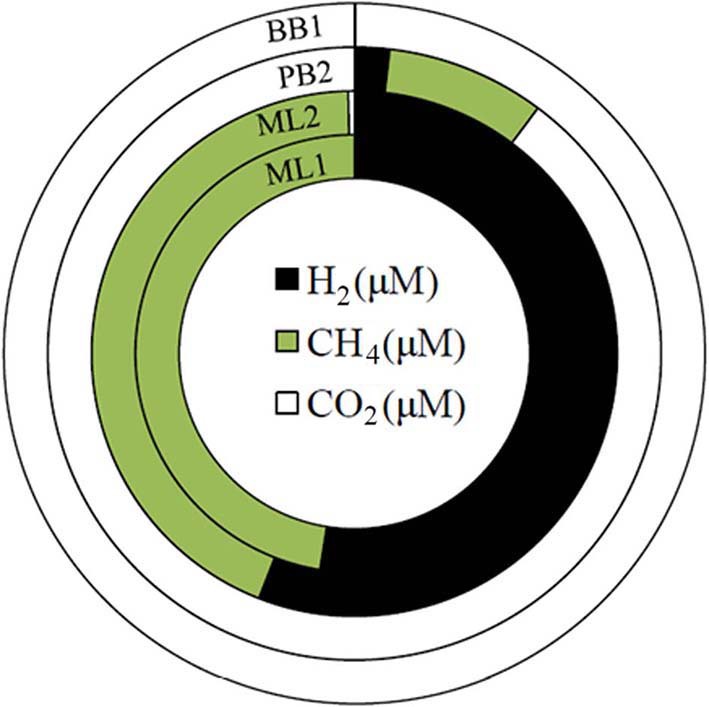
**Dissolved gas findings (reported in Table 3) shown as side-by-side gas doughnut diagrams**. Note that the sampled sites range from H_2_- and CH_4_-dominated (ML1 and ML2, inner green and black doughnuts) to nearly completely CO_2_-dominated (BB1, white doughnut).

Waters can be grouped into four types based on dissolved gas constituents. ML1 and PB3 bear CO, CH_4_, and H_2_. ML2, PB2, and SI1 bear CO_2_, CH_4_, and H_2_. BB1 is rich in CO_2_ only. MF1 had overall low gas contents, although CH_4_ was observed. Taken together, these springs cover a range in gas contents (Table 3, Figure 6).

**Table 3:** **Dissolved gas concentrations, obtained via gas-stripping of gas-rich aqueous samples**.

**Locality**	**Site**	**H_2_(μM)**	**CO(μM)**	**CH_4_(μM)**	**CO_2_(μM)**
Zambales, 2012	ML1	207.0	0.1	187.2	0.0
	ML2	239.1	0.0	186.8	1.6
	ML3	0.0	0.0	0.0	0.0
	BB1	0.0	0.1	1.2	2568.2
	PB1	0.0	0.0	0.0	0.0
	PB2	7.2	0.2	33.5	359.4
	PB3	0.0	0.0	0.0	0.0
	PBR	0.0	0.0	0.0	0.0
Palawan, 2012	SI1	0.0	0.1	631.6	57.5
	MF1	–	–	–	–
Zambales, 2013	ML1	473.9	0.0	372.8	23.9
	ML2	495.5	0.0	400.0	6.8
	ML3	85.4	0.0	119.8	120.2
	BB1	0.0	0.0	0.0	2496.4
	PB1	161.2	0.0	BDL	BDL
	PB2	0.0	0.0	BDL	1755.3
	PBR	–	0.0	–	–

Given that (a) gas contributions from a subsurface zone undergoing active serpentinization is expected to yield H_2_ and CH_4_ and (b) extremely high pH, Ca^2+^-OH^−^ waters are inherently poor in aqueous CO_2_ since they have been isolated from the atmosphere, and are thus poor in DIC, first order conclusions can be drawn from Figure 6. Dissolved gases at Bigbiga (BB1) consist essentially of CO_2_. This spring stands aparts from the others as a CO_2_-effervescing spring with relatively oxidized gas phase carbon, and no detected reduced gases. It is likely open to the atmosphere at some point in its recharge path. H_2_ and CH_4_ are observed at low concentrations at site PB2, with the gas balance as CO_2_. At PB2, the data can be interpreted as reflecting mixing between a CO_2_-bearing, surface-derived, shallow groundwater regime and a separate, deeply sourced water carrying reduced gases. ML1, ML2 are nearly 50%—50% mixtures of H_2_ and CH_4_, with a small proportion of CO_2_, if present. Gas contents at ML1 and ML2 indicate that these waters provide a plausible, deeply sourced end-member composition.

## Discussion

### Aqueous and gas geochemistry

Of these newly characterized sites in the Philippines Ophiolites, the Poon Bato springs PB1 and PB3 are typical Ca^2+^-OH^−^ type waters (c.f., Barnes et al., 1967), and can be interpreted as sourced in actively serpentinizing host rock; PB2 has relatively higher Mg^2+^ content, indicative of some mixing with open system Mg^2+^-HCO^−^_3_ waters. The Manleluag springs ML1–ML3 express a muted serpentinization signal in the aqueous geochemistry, with less Ca^2+^ and lower pH. Given that all country rock observed in the Manleluag area was gabbroic (deep ocean crustal rocks rather than peridotitic mantle units), waters may be escaping along a subsurface mafic-ultramafic contact (i.e., the interface between the gabbros and altering peridotites, or the interface between the crust and the mantle rocks). The gas data for PB and ML sites support this interpretation, indicating incorporation of H_2_ and CH_4_ likely derived from regional serpentinization.

BB1 well water samples, known to be sourced in altered pillow basalts may have some hydrological communication with extensive, adjacent bentonite clay sedimentary deposits. These waters have a lower pH signal, higher salinity, and are the only waters in which sulfate was detected in both sampling years, perhaps due to perennial leakage from sedimentary formations. Note the changing scale in Figure 4, with increasing salinity for BB1; SI1 and MF1 waters also have enhanced salinity, dramatically so for MF1, with range up to 12 meq/kg in Figure 4, along with relatively reducing oxidation-reduction potential. The relatively high salinity coupled with the weak Ca signal suggests that these waters (MF1, SI1) are groundwaters isolated from serpentinization reactions in the subsurface.

Variation in DIC and DOC between sites and at the same sites in different years is unpredictable (Table 2). These field locations are some of the first described in tropical climates, and are likely heavily influenced not only by seasonal variations in precipitation, but also vegetative input to the springs. As some site locations were sampled in different years, and under different climate conditions, these unpredictable variations can be observed (Table 4). DIC is typically low in sites with corresponding low ORP, and high in locations that have more opportunity to equilibrate with atmospheric CO_2_ (e.g., BB1, PBR, MF1). Comparing 2012 vs. 2013 values, which represent dryer vs. wetter climate conditions, respectively, concentrations of DIC increase at some sites and decrease at others. Presumably, the underlying hydrology in combination with surface influences cause these variations. The isotopic composition of DIC in these fluids ranges from ~ −11 to −25‰. Values that approach ~ −7‰ likely indicate interactions with atmospheric CO_2_, in other words, an open system with respect to CO_2_, while more depleted values may reflect microbial waste product build up in springs that are somewhat isolated from surface processes. In comparison to other terrestrial serpentinizing seeps (few, where data are available), DIC at most Philippines sites have enriched isotopic compositions. For example, The Cedars (CA, USA) and Tablelands (Canada) locations host fluids with DIC that show isotopic compositions between ~ −30 to −32‰ (Morrill et al., 2013; Szponar et al., 2013). Again, DIC that is isotopically enriched may indicate mixing with fluids that are more associated with surface processes. Regardless, low DIC concentrations in the Philippines systems mean that subsurface microbial ecosystems (1) may be carbon poor, and (2) any microbial fractionation during carbon fixation will be starting from carbon that is less enriched in ^13^C than surface fluids. This latter will have implications for any additional studies of carbon isotope compositions of biomass.

**Table 4:** **New data for the stable isotopes of water in springs of the Zambales and Palawan Ophiolites, presented with additional regional data, related to the establishment of a local meteoric water line (Gerardo-Abaya, 2005) and documentation of springs with isotopic characteristics signaling interaction with country rock and magmatic processes (Giggenbach, 1992)**.

**Site**	**δ^18^O (SMOW)**	**δ D (SMOW)**	**References**
Zambales, PB1	−8.4	−55.8	This study
Zambales, PB2	−8.5	−57.2	This study
Zambales, ML1	−9.4	−60.9	This study
Zambales, ML2	−9.3	−60.7	This study
Zambales, BB1	−7.9	−55.5	This study
Palawan, SI1	−7.5	−45.6	This study
Palawan, MF1	−6.9	−43.6	This study
Quezon City, NIGS, meteoric water	−7.4	−52.8	This study
Southern Negros Geothermal Field, shallow groundwater	−7.2	−45.0	Gerardo-Abaya, 2005
Southern Negros Geothermal Field, deep groundwater	−7.4	−46.5	Gerardo-Abaya, 2005
Southern Negros Geothermal Field, warm springs	−7.1	−48.0	Gerardo-Abaya, 2005
Southern Negros Geothermal Field, cold springs	−7.5	−48.0	Gerardo-Abaya, 2005
Alto Peak, meteoric water	−7	−44	Giggenbach, 1992
Alto Peak, most shifted andesitic water	2.6	−22	Giggenbach, 1992
Cagua, meteoric water	−7.5	−50	Giggenbach, 1992
Cagua, most shifted andesitic water	4.5	−27	Giggenbach, 1992
Mahagnao, meteoric water	−7.5	−50	Giggenbach, 1992
Mahagnao, most shifted andesitic water	8	−20	Giggenbach, 1992
Mt. Pinatubo, meteoric water	−8	−54	Giggenbach, 1992
Mt. Pinatubo, most shifted andesitic water	5	−29	Giggenbach, 1992

DOC concentrations are similarly unpredictable, between sample locations and sample years. The degree of additional organic carbon input from the surrounding heavily vegetated surface environment is a variable that cannot be controlled. Thus, DOC may play a large role in microbial metabolic function in these particular springs that other terrestrial serpentinizing springs lack (such as those in more arid climates, for example), even though the overall concentration of DOC in these systems appears to be relatively low. It might be expected that DOC concentrations would increase with increased precipitation. However, with only a few exceptions, this is not the case in the Philippines field sites. In contrast, increased precipitation may have diluted the DOC, or contributed to otherwise flushing vegetative build up from the pools. Isotopic composition of the DOC is, predictably, depleted relative to that of DIC at all sites except for the 2012 PB1 sample. DOC in other terrestrial serpentinizing systems has also been reported to be low (<2 ppm—Morrill et al., 2013; Szponar et al., 2013). However, the Philippines systems may have benefit of a more consistently supplied influx of surface derived DOC, due to the dense vegetation and frequent flushing (and refreshing) of the system during precipitation events. DOC isotopic compositions at these sites are more depleted in comparison with DOC reported from the Tablelands and the Cedars locations (Morrill et al., 2013; Szponar et al., 2013)—this may indicate less recycling of carbon in the system compared to other terrestrial sites. Further, heterotrophic metabolism in the subsurface should produce isotopically heavier CO_2_ as a waste product, which would be added to any native DIC.

### Geochemical modeling of metabolic reactions

**Figure 7:**
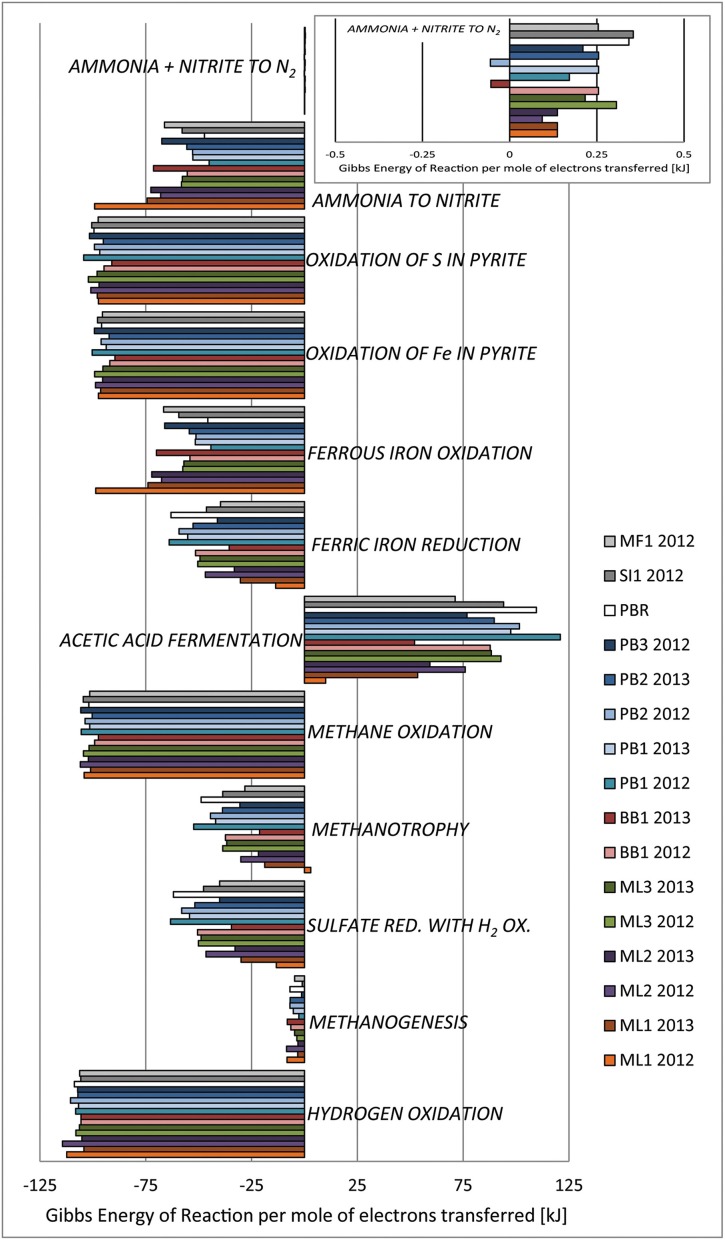
**Microbiological metabolic strategies, pitched as chemical reactions, are evaluated for Gibbs energy yield as in Amend and Shock (2001), based on modeled activities derived from environmental data**. Recall that spontaneous reactions have Δ*G_r_* < 0. The geochemical environments considered here cause shifts in the thermodynamic feasibility of reactions. TDS were estimated from field conductivity data. Hydrogen oxidation: H_2(aq)_ + 0.5O_2(aq)_ = H_2_O_(l)_ Methanogenesis: CO_2(aq)_ + 4H_2(aq)_ = CH_4(aq)_ + 2H_2_O_(l)_ Sulfate reduction coupled to hydrogen oxidation: SO^2−^_4_ + 4H_2(aq)_ +2H^+^ = H_2_S_(aq)_ + 4H_2_O_(l)_ Methanotrophy: CH_4(aq)_ + SO^2−^_4_ + 2H^+^ = HCO^−^_3_ + H_2_S_(aq)_ + H_2_O_(l)_ Methane oxidation: CH_4(aq)_ + 2O_2(aq)_ = CO_2(aq)_ + 2H_2_O_(l)_ Acetic acid disproportionation: acetic acid(aq) = CH_4(aq)_ + CO_2(aq)_ Ferric iron reduction: H_2(aq)_ + 2Fe^3+^ = 2Fe^2+^ + 2H^+^ Ferrous iron oxidation: 2Fe^2+^ + 0.5 O_2(aq)_ + 2H^+^ = 2Fe^3+^ + H_2_O_(l)_ Oxidation of Fe in pyrite: 2 pyrite(s) + 7.5O_2(aq)_ + H_2_O_(l)_ = 2Fe^3+^ + 4SO^2−^_4_ + 2H^+^ Oxidation of S in pyrite: pyrite + 3.5O_2(aq)_ + H_2_O_(l)_ = Fe^2+^ + 2SO^2−^ + *2H^+^* Nitrification: NH_3_ + 1.5O_2(aq)_ = H^+^ + NO^−^_2_ + H_2_O_(l)_ Ammonia oxidation: NH_3(aq)_ + NO^−^_2_ + H^+^ = N_2(aq)_ + 2H_2_O_(l)_.

We modeled the activities of chemical species important for microbial metabolisms based on geochemical data for 9 ophiolite-associated groundwaters (several sampled in both field seasons) and 1 regional river water, to determine if selected metabolic reactions are supported in these aqueous environments. Metabolisms (reactions provided in caption to Figure 7) were chosen to mirror those chemosynthetic metabolic strategies known to be utilized at sites of active serpentinization (such as those profiled in Table 1). For reference, Schrenk et al. (2013) group continental and marine serpentinites conceptually as a biome, detailing that low cell counts and a relatively low diversity of microbial types have been documented in a number of field studies in serpentinites. In the Archaea, representatives of the Crenarchaeota (Desulfurococcales) and Euryarchaeota, (Archaeoglobales, Thermococcales, Methanococcales, Methanobacteriales, Methanopyrales, Methanosarcinales, ANME-2, and ANME-1 groups) have been detected. In the Bacteria, representatives of the Aquificae, Bacteroidetes, Betaproteobacteria (including Hydrogenophaga), Gammaproteobacteria, Deltaproteobacteria, Epsilonproteobacteria, Thermodesulfobacteria, Actinobacteria, and Firmicutes have been observed (Schrenk et al., 2013). Accordingly, metabolisms that are tied to methane cycling and hydrogen oxidation are considered here, as well as other reactions that make use of ferric iron, sulfur, and/or N-compounds in the ultramafic environment.

Hydrogen oxidation via the knallgas reaction has the greatest bioenergetic yield in the modeled system. In general, given the nature of this study, environmental samples were collected at the interface between a relatively reducing aqueous system (groundwaters linked to serpentinization in most cases) and a relatively oxidizing system (open to the atmosphere). Although energy yields are not as great for sulfate reduction coupled to hydrogen oxidation, where sulfate is of meaningful concentration (BB1, ML sites, MF1, SI1), this metabolism is also feasible.

Several aspects of methane cycling were treated in this model. Because spring waters and well samples were all collected at the interface between surface and subsurface biogeochemical systems, it is to be expected that CO_2_ is dissolving into the system, providing the reactants for methanogenesis, and it is to be expected that organic acids produced abiotically by serpentinization would also be present, possibly fueling acetic acid disproportionation. Both of these metabolisms were evaluated in the model. Methanogenesis is feasible but with the lowest calculated energy yield. Acetic acid fermentation is not feasible, likely due to the environmental abundance of methane and very low expected concentrations of acetate. Methane is abiotically produced in serpentinization, and so would be a common reactant for metabolism: both methane oxidation and methanotrophy coupled with sulfate reduction are feasible based on the model results.

Given the possible mixing of Type I and II waters in these subsurface hydrological flow regimes, there may be aqueous ferrous iron inputs tied to the surface-related water; at the same time, the mineral-hosted iron in serpentinized ultramafics is likely to be ferric, with some ferrous iron locked in nearly insoluble oxides and oxyhydroxides. In the absence of thermodynamic data at elevated temperatures for many solid phases of interest in microbial iron cycling (such as the minerals goethite and ferrihydrite), we relied on the aqueous phase Fe redox transformations, coupled with H_2_ oxidation: both iron oxidation and reduction are feasible under the modeled environmental conditions.

Pyrite biodegradation, through the oxidation of either iron or sulfur, is feasible under the modeled conditions also, with relatively high energy yields. Indeed, serpentinization has been considered an important sulfur sink in the seabed (Alt and Shanks, 1998) and pyrite may be pervasive in the subsurface at ML and PB sites. In the case of BB1, we sampled an artesian well plumbing groundwaters interacting with altered pillow basalts and clay-rich sedimentary formations. The adjacent sedimentary unit is composed primarily of smectite-group clays and zeolites (x-ray diffraction data are not shown, but confirm mineral identifications), and pyrite is a reasonable minor component in this system, as a common diagenetic mineral.

Nitrification and ammonia oxidation reactions were also assessed for bioenergetics yield. Nitrification was thermodynamically favored in the modeled environments, while ammonia oxidation was not. Ammonia oxidation, as written here (Figure 7 caption) requires protons as a reactant, and in the high pH environments, the dearth of protons likely impedes this reaction. In fact, the energy yields are near zero for all sites considered. However, the inset box in Figure 7 shows the ammonia oxidation results hover near Δ*G_r_* = 0; note that the Gibbs energy values for this metabolic strategy are slightly negative (thus the metabolism is favored) only for sites BB1 and PB2 in 2013. The swing from Δ*G_r_* > 0 to Δ*G_r_* < 0 between sampling years indicates that the metabolic strategy may be feasible under some field conditions, and does in fact shift from environmentally supported to environmentally inhibited over time, as is shown by our year-to-year shift.

In sum, calculated Gibbs energy of reaction values (calculated at environmental conditions) at all sites indicate that most selected metabolisms are indeed feasible, with Gibbs energy <0, indicating a thermodynamic potential for the reaction to proceed to the right, as written, under the specified conditions (Figure 7). Two exceptions occur: acetic acid disproportionation [i.e., acetic acid_(aq)_ = CH_4(aq)_ + CO_2(aq)_[ and ammonia oxidation in the presence of nitrite [i.e., NH_3(aq)_ + NO^−^_2_ + H^+^ = N_2(aq)_ + 2H_2_O_(l)_] are not shown to be feasible in the geochemical environment. No site supports acetic acid disproportionation (taken as a proxy for fermentation of acetate).

Interestingly, working with environmental samples collected at ML sites in tandem with those used for geochemical analysis and thus linked directly to the model results, Woycheese et al. (2015) report several putative metabolisms (based on nearest neighbor taxonomic affiliations via 16S rRNA gene amplicon sequencing on the Illumina MiSeq platform) that resonate with the geochemical modeling presented here.

Hydrogen oxidation is likely a major metabolism in the ML series springs: 16S data show that there are two hydrogen-oxidizing genera in the environment. *Hydrogenophaga* comprised 1.3% of sequence reads at the upstream edge of site ML2, 7.1% of reads at the downstream edge of ML2, and 3.4% of reads at ML3. *Thiobacillus* (family *Hydrogenophilace*) accounted for 0.02, 4.4, and 9.2% of sequence reads, at ML1–ML3, respectively.

16S data also inform the case for methane cycling in these ophiolite-associated springs. Methanogenesis is likely one of the dominant metabolisms at ML1: the archaeal family *Methanobacteriaceae* makes up about 7% of sequence reads at the upstream edge of site ML2, and 3.4% of reads at the downstream edge of ML2. At ML3, percentages are much lower (0.06% of reads); suggesting communities shift as the physical-chemical conditions evolve in the running stream water. We noted the methanotrophic bacterial genera *Methylobacteriaceae*, *Methylocystaceae*, and *Methylobacillus* in the rare taxa, comprising less than 0.03% of sequence reads. Acetic acid fermentation is not prominent in the 16S data, but there are traces of the family *Acetobacteraceae* at the upstream edge of site ML2 (0.01% sequence abundance), downstream edge of site ML2 (0.4% sequence abundance), and ML3 (1.2% sequence abundance). 16S data do not speak to sulfur cycling, and have very little evidence for iron cycling in the ML sites. Here, less than 0.2% of sequence reads aligned with the iron-oxidizing bacterial family *Thermodesulfovibrionaceae*. Lastly, ammonia-related sequence reads are not significant; the archaeal genus *Candidatus Nitrososphaera* comprised less than 0.099% of sequence reads for ML sites.

## Concluding remarks

In this work, we construct one possible model for the metabolic niches available to microbes in several ophiolite-associated springs in the Zambales and Palawan Ophiolites, Philippines. We provide field descriptions, new aqueous environmental data, and an analysis of the feasibility of several microbial metabolisms that are prominent in deep subsurface habitats. We find that several springs (ML series and PB series) exhibit geochemical patterns indicative of active serpentinization in the ultramafic subsurface, while waters sampled at BB1, SI1, and MF1 appear to be mixtures of meteoric and other waters derived from the alteration of other units in the oceanic crust, now tectonically emplaced on land. The composite metabolic framework observed in these ophiolite-hosted springs, presented graphically here (Figure 7), aligns some hydrogen-oxidizing, methane cycling, Fe- and S-utilizing, and nitrogen cycling reactions, illustrating metabolisms available to subsurface microbes. This work defines a metabolic space or “landscape” based on system geochemistry, and provides a framework in which to conduct analyses of active metabolic strategies in this geologically unique environment.

### Conflict of interest statement

The authors declare that the research was conducted in the absence of any commercial or financial relationships that could be construed as a potential conflict of interest.
