# Supplementary material for: Feasible metabolisms in high pH springs of the Philippines
Source: Front Microbiol. 2015 Feb 10;6:10. doi: 10.3389/fmicb.2015.00010 (PMC4322734; doi:10.3389/fmicb.2015.00010)
Supplement: Supplementary file 1 [file DataSheet1.PDF]

DATA SHEET 1. EQ3 Modeling inputs.

Site	Temp. (°C)	TDS (ppm)	Eh (V)	pH	Na^+ (ppm)	K^+ (ppm)	Ca^{2+} (ppm)	Mg^{2+} (ppm)	Fe^{2+} (ppm)	HCO_3^- (ppm)	Cl^- (ppm)	SO_4^{2-} (ppm)	NO_3^- (ppm)	$CH_{4(aq)}$ (M)	$H_{2(aq)}$ (M)	$O_{2(aq)}$ (mg/L)
ML1 2012	34.4	202	-0.503	10.9	23	0.2	3.1	0.001	0.02	0.6	18.0	5.0	4.1	0.000187	0.000207	0.1
ML1 2013	34.4	248	-0.260	10.9	18.5	0.3	8.4	0.2	0.039	0.9	17.0	0.1	4.0	0.000001	0.000001	0.014
ML2 2012	34.0	216	-0.155	10.8	24.4	0.2	3.9	0.001	0.02	0.5	18.7	0.7	4.3	0.000187	0.000239	1.88
ML2 2013	34.4	248	-0.225	10.8	18.7	0.3	6.0	0.001	0.027	0.4	16.9	18.5	1.6	0.000001	0.000001	0.063
ML3 2012	33.8	196	-0.045	10.8	22.6	0.20	3.3	0.1	0.02	1.5	17.0	0.8	5.0	0.000001	0.000001	4.41
ML3 2013	32.6	173	-0.026	10.3	19.8	0.4	2.2	1.7	0.032	4.4	9.8	19.3	0.1	0.000001	0.000001	0.311
BB1 2012	29.7	223	0.054	9.3	100.5	0.5	1.5	0.001	0.02	37.0	4.4	47.3	1.7	0.000001	0.000001	0.061
BB1 2013	28.0	274	0.026	7.0	119.9	0.7	1.1	0.001	0.02	35.6	5.0	46.6	1.1	0.000001	0.000001	0.047
PB1 2012	31.5	323	0.064	11.3	23.9	1.3	52.8	0.001	0.3	1.3	24.0	0.1	1.3	0.000001	0.000001	4.413
PB1 2013	29.7	148	0.075	9.6	19.2	0.5	37.2	0.1	0.6	3.0	12.4	9.6	1.4	0.000001	0.000001	0.418
PB2 2012	27.2	147	0.114	9.2	10.3	0.6	8.0	7.6	0.02	1.0	11.3	0.1	0.9	0.000034	0.000007	2.571
PB2 2013	29.7	121	0.103	8.7	14.8	0.2	12.9	20.7	0.02	22.5	10.9	89.1	1.5	0.000001	0.000001	0.661
PB3 2012	28.6	388	-0.173	11.3	15.6	0.8	50.2	0.2	0.02	1.0	17.9	0.1	0.1	0.000108	0.000001	0.611
PBR 2012	27.9	115	0.215	8.6	1.4	0.1	2.0	23.3	0.02	18.5	7.3	0.1	0.1	0.00001	0.000001	7.66
SI1 2012	47.7	330	-0.065	10.5	91.6	0.7	3.6	0.001	0.02	3.9	57.4	3.9	7.5	0.000632	0.000001	0.682
MF1 2012	40.6	502	-0.087	9.7	269.7	4.5	2.5	0.001	0.02	28.1	228.7	9.7	0.1	0.000006	0.000001	0.665
