# Supplementary material for: Feasible metabolisms in high pH springs of the Philippines
Source: Front Microbiol. 2015 Feb 10;6:10. doi: 10.3389/fmicb.2015.00010 (PMC4322734; doi:10.3389/fmicb.2015.00010)
Supplement: Supplementary file 2 [file Table1.DOCX]

Supplementary Materials

Table S1. EQ3 Modeling inputs

| Site | Temp. (°C) | TDS (ppm) | Eh (V) | pH | Na^+^ (ppm) | K^+^  (ppm) | Ca^2+^  (ppm) | Mg^2+^  (ppm) | Fe^2+^  (ppm) | HCO_3_^-^(ppm) | Cl^-^(ppm) | SO_4_^2-^(ppm) | NO_3_^-^(ppm) | CH_4(aq)_  (M) | H_2(aq)_  (M) | O_2(aq)_  (mg/L) |
| --- | --- | --- | --- | --- | --- | --- | --- | --- | --- | --- | --- | --- | --- | --- | --- | --- |
| ML1 2012 | 34.4 | 202 | -0.503 | 10.9 | 23 | 0.2 | 3.1 | 0.001 | 0.02 | 0.6 | 18.0 | 5.0 | 4.1 | 0.000187 | 0.000207 | 0.1 |
| ML1 2013 | 34.4 | 248 | -0.260 | 10.9 | 18.5 | 0.3 | 8.4 | 0.2 | 0.039 | 0.9 | 17.0 | 0.1 | 4.0 | 0.000001 | 0.000001 | 0.014 |
| ML2 2012 | 34.0 | 216 | -0.155 | 10.8 | 24.4 | 0.2 | 3.9 | 0.001 | 0.02 | 0.5 | 18.7 | 0.7 | 4.3 | 0.000187 | 0.000239 | 1.88 |
| ML2 2013 | 34.4 | 248 | -0.225 | 10.8 | 18.7 | 0.3 | 6.0 | 0.001 | 0.027 | 0.4 | 16.9 | 18.5 | 1.6 | 0.000001 | 0.000001 | 0.063 |
| ML3 2012 | 33.8 | 196 | -0.045 | 10.8 | 22.6 | 0.20 | 3.3 | 0.1 | 0.02 | 1.5 | 17.0 | 0.8 | 5.0 | 0.000001 | 0.000001 | 4.41 |
| ML3 2013 | 32.6 | 173 | -0.026 | 10.3 | 19.8 | 0.4 | 2.2 | 1.7 | 0.032 | 4.4 | 9.8 | 19.3 | 0.1 | 0.000001 | 0.000001 | 0.311 |
| BB1 2012 | 29.7 | 223 | 0.054 | 9.3 | 100.5 | 0.5 | 1.5 | 0.001 | 0.02 | 37.0 | 4.4 | 47.3 | 1.7 | 0.000001 | 0.000001 | 0.061 |
| BB1 2013 | 28.0 | 274 | 0.026 | 7.0 | 119.9 | 0.7 | 1.1 | 0.001 | 0.02 | 35.6 | 5.0 | 46.6 | 1.1 | 0.000001 | 0.000001 | 0.047 |
| PB1 2012 | 31.5 | 323 | 0.064 | 11.3 | 23.9 | 1.3 | 52.8 | 0.001 | 0.3 | 1.3 | 24.0 | 0.1 | 1.3 | 0.000001 | 0.000001 | 4.413 |
| PB1 2013 | 29.7 | 148 | 0.075 | 9.6 | 19.2 | 0.5 | 37.2 | 0.1 | 0.6 | 3.0 | 12.4 | 9.6 | 1.4 | 0.000001 | 0.000001 | 0.418 |
| PB2 2012 | 27.2 | 147 | 0.114 | 9.2 | 10.3 | 0.6 | 8.0 | 7.6 | 0.02 | 1.0 | 11.3 | 0.1 | 0.9 | 0.000034 | 0.000007 | 2.571 |
| PB2 2013 | 29.7 | 121 | 0.103 | 8.7 | 14.8 | 0.2 | 12.9 | 20.7 | 0.02 | 22.5 | 10.9 | 89.1 | 1.5 | 0.000001 | 0.000001 | 0.661 |
| PB3 2012 | 28.6 | 388 | -0.173 | 11.3 | 15.6 | 0.8 | 50.2 | 0.2 | 0.02 | 1.0 | 17.9 | 0.1 | 0.1 | 0.000108 | 0.000001 | 0.611 |
| PBR 2012 | 27.9 | 115 | 0.215 | 8.6 | 1.4 | 0.1 | 2.0 | 23.3 | 0.02 | 18.5 | 7.3 | 0.1 | 0.1 | 0.00001 | 0.000001 | 7.66 |
| SI1 2012 | 47.7 | 330 | -0.065 | 10.5 | 91.6 | 0.7 | 3.6 | 0.001 | 0.02 | 3.9 | 57.4 | 3.9 | 7.5 | 0.000632 | 0.000001 | 0.682 |
| MF1 2012 | 40.6 | 502 | -0.087 | 9.7 | 269.7 | 4.5 | 2.5 | 0.001 | 0.02 | 28.1 | 228.7 | 9.7 | 0.1 | 0.000006 | 0.000001 | 0.665 |


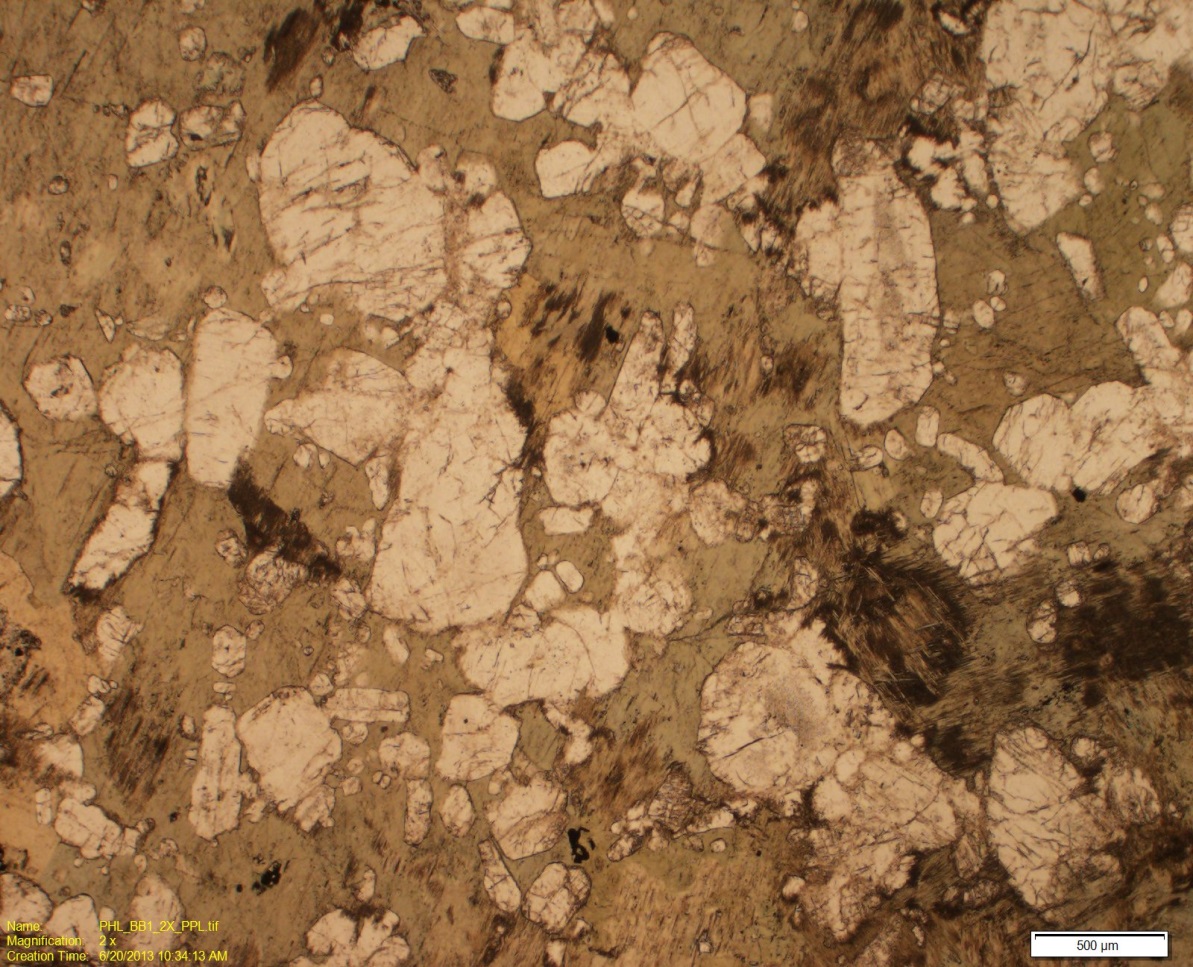

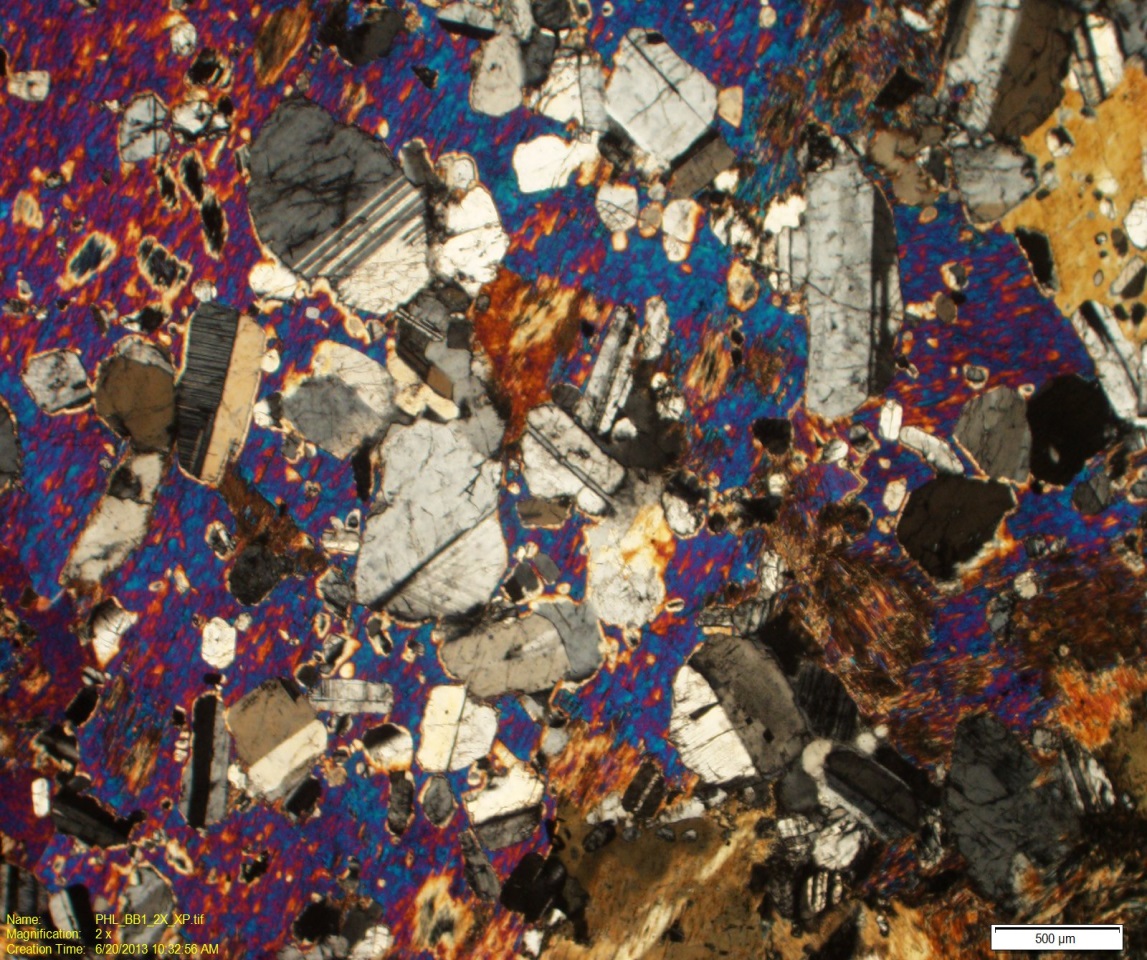


**D**

**E**
